# First report of a post-pneumonectomy nocardia empyema

**DOI:** 10.1093/jscr/rjae002

**Published:** 2024-01-30

**Authors:** Jonathan A Nakata, Azzan Arif, Louis F Chai, Stacey Su

**Affiliations:** Division of Thoracic Surgery, Department of Surgical Oncology, Fox Chase Cancer Center, Philadelphia, PA 19111, United States; Division of Thoracic Surgery, Department of Surgical Oncology, Fox Chase Cancer Center, Philadelphia, PA 19111, United States; Department of Thoracic Medicine and Surgery, Temple University Health Systems, Philadelphia, PA 19140, United States; Division of Thoracic Surgery, Department of Surgical Oncology, Fox Chase Cancer Center, Philadelphia, PA 19111, United States; Department of Thoracic Medicine and Surgery, Temple University Health Systems, Philadelphia, PA 19140, United States

**Keywords:** Nocardia, Nocardia nova, empyema, pneumonectomy, post-pneumonectomy empyema, PPE, chest infection, VATS, immunocompetent, lung cancer, NSCLC, adenocarcinoma

## Abstract

Post-pneumonectomy empyema (PPE) is an uncommon but serious complication that carries significant therapeutic challenges. We present a late-onset PPE due to *Nocardia nova* in an immunocompetent individual. Nine years after a right pneumonectomy for non-small cell lung cancer, surveillance scans revealed new right pleural thickening and FDG avidity concerning for recurrence. Thoracoscopic pleural biopsies were negative for malignancy, but tissue cultures grew *N. nova*. Nocardia empyema is rare with few reported cases. Most occur in immunocompromised hosts, and all were associated with pulmonary or disseminated nocardiosis. Our case describes the first report of a PPE secondary to Nocardia.

## Introduction

Post-pneumonectomy empyema (PPE) occurs with a range of 2–16% with most reporting an incidence of 5% [1–6]. Development of empyema in a pneumonectomy space can be classified as early or late. 60–75% of empyemas occur within three months after pneumonectomy [4, 6]. Late-onset PPE occurs greater than 3 months post-operatively and have occurred up to 40 years following surgery [3–5]. In the current literature, few cases of Nocardia empyema have been described, but none have occurred post-pneumonectomy. We present the first report of a post-pneumonectomy Nocardia empyema.

### Case report

**Figure 1:**
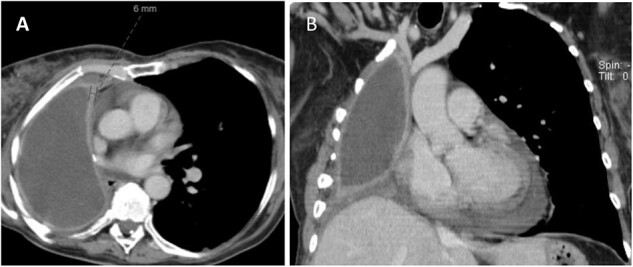
CT (A) axial and (B) coronal views showing fluid filled pneumonectomy space with nodularity and thickened right pleura measuring 6 mm anteriorly.

**Figure 2:**
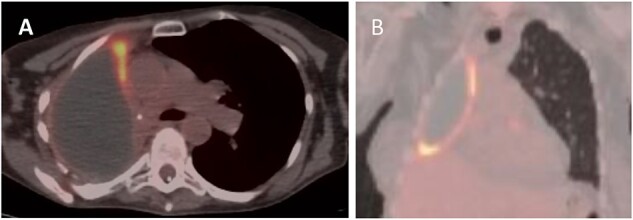
PET (A) axial and (B) coronal views showing increased FDG uptake (max SUV 9.5) along the anteromedial and inferior aspects of the right lung pleura.

**Figure 3:**
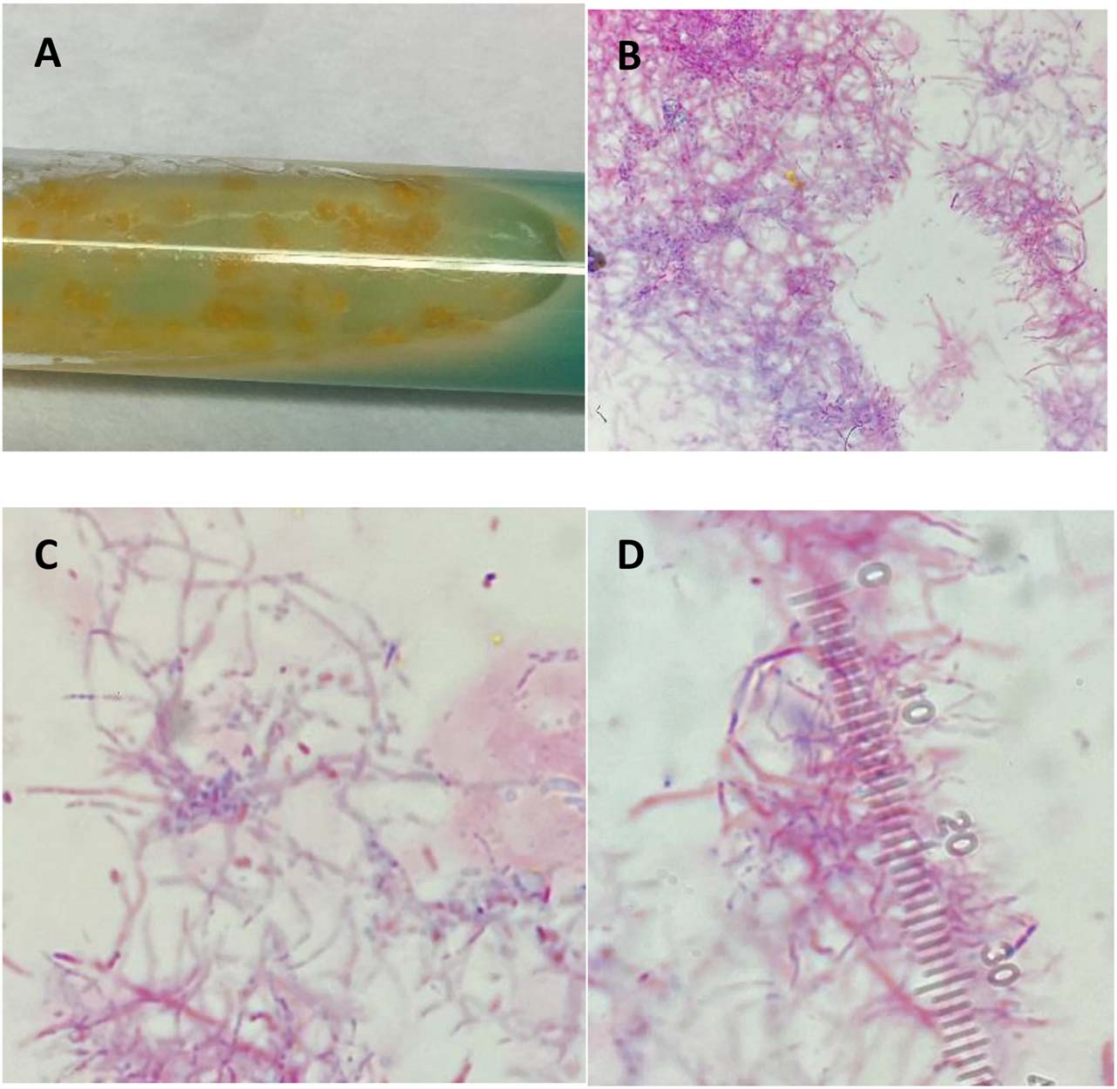
(A) Colonies grown in broth medium using modified Kinyoun stain. (B–D) Light microscopy features filamentous branching rods stained acid fast positive due to the mycolic acid present in cell walls.

A 60-year-old woman with stage IIIA right lung adenocarcinoma underwent neoadjuvant chemoradiation followed by pneumonectomy. Nine years later, surveillance CT/PET scans revealed new right pleural thickening and nodular avidity with concerns for malignant recurrence (Figs 1 and 2). Her only symptom was chronic chest pain attributed to post-thoracotomy syndrome. She had no respiratory symptoms. She underwent a bronchoscopy which showed an intact right mainstem bronchial stump without signs of tumor or fistula. Upon entry into the pleural cavity during video-assisted thoracoscopic surgery for pleural biopsy (VATS), 10 mL of white, non-odorous fluid was drained. There was no intra-operative suggestion of cancer recurrence. Pathology of the pleural biopsy showed acute inflammation with underlying chronic pleuritis and no tumor. Pleural cultures were positive for *N. nova*, consistent with PPE (Fig. 3). She then started on intravenous trimethoprim-sulfamethoxazole and amikacin. Brain MRI and blood cultures were negative for disseminated nocardiosis. She was planned for washout and muscle transposition to fill the pneumonectomy space, but clinically deteriorated after aspiration pneumonia and cardiogenic pulmonary edema. She elected for comfort measures and expired shortly afterwards.

## Discussion

PPE is a serious complication with a mortality of 25%, increasing to 50% when associated with a bronchopleural fistula (BPF) [6]. Common causes of death are pneumonia, ARDS and sepsis [2, 4]. The mechanism of PPE development can be characterized by time of onset [3]. Early PPE is caused by direct contamination of the pneumonectomy space intraoperatively or by seeding from BPFs or esophageal-pleural fistulas. Late PPEs typically develop by hematogenous spread from a distant infectious source or by delayed BPF formation. Multiple risk factors have been identified including diabetes, poor nutrition, low preoperative pulmonary function tests, neoadjuvant therapy, long bronchial stump, positive resection margins and right pneumonectomy [1, 4, 5]. Our patient’s known risk factors were neoadjuvant chemoradiation and right-sided pneumonectomy.

Diagnosis can be challenging as the onset is often insidious and can occur years after the index operation. The average time to diagnose late PPE usually exceeds 1 month after presentation [1, 3]. Symptoms are non-specific such as low-grade fever, chills, weight loss, anorexia, weakness and myalgias [3]. Other manifestations can include empyema necessitans, expectoration of purulent or serosanguineous fluid when associated with BPF, or drop in air-fluid level on chest X-ray [2]. Our patient’s only symptom was progressive chest pain, which may have been secondary to Nocardia PPE rather than the presumed post-thoracotomy pain syndrome.

Polymicrobial infections occur in 49% of cases but are more common in early PPE (65%) versus late PPE (25%) [3, 5]. Organisms most commonly isolated in PPE are Staphylococcus, Pseudomonas and Streptococcus in otherwise immunocompetent individuals [1, 2, 4]. Conversely, Nocardia empyema is exceedingly rare with all reported cases associated with pulmonary or disseminated nocardiosis, particularly in immunocompromised patients. A search of the PubMed/MEDLINE database using keywords ‘pneumonectomy’, ‘empyema’ and ‘Nocardia’ found no report of PPE due to *Nocardia*. Nocardia is a ubiquitous, aerobic, gram-positive, branching and weakly acid-fast bacillus found in soil [7–10]. Transmission occurs from inhalation and often affects the lungs [8]. There is propensity for hematogenous dissemination, most commonly to the brain [7, 8]. Treatment for nocardiosis relies on an extended course of parenteral antibiotics regimens depending on susceptibility [9, 10].

The treatment of PPE remains challenging with multiple options including tube thoracostomy with irrigation, VATS/thoracotomy washout and drainage, or window thoracostomy with muscle transposition into the pleural space [1–6]. Although our patient expired prior to final treatments, plans for washout with flap coverage followed by a 1-year antibiotic course were intended. It remains unclear how the pneumonectomy space became infected with Nocardia in this patient. There was no evidence of BPF, no signs of disseminated nocardiosis, nor was she immunocompromised. Our case highlights the insidious nature of PPE and the importance of microbial analysis to ensure adequate treatment.

PPE is a devastating complication following pneumonectomy. Clinicians should be familiar with timing of PPE development and recognize disease potential despite non-specific symptoms. Thorough evaluation is required for prompt diagnosis and intervention in cases with uncommon etiologies.
